# *catena*-Poly[[[diaqua­diformato­cobalt(II)]-μ-1,4-bis­(1*H*-benzimidazol-1-yl)benzene] dihydrate]

**DOI:** 10.1107/S160053681105505X

**Published:** 2012-01-07

**Authors:** Ping-Yun Huang, Jin-Guo Wang, Sheng-Wu Guo, Gang Shi

**Affiliations:** a College of Science, Chang’an University, Xi’an 710064, Shaanxi, People’s Republic of China; b Key Laboratory for Mechanical Behavior of Materials, Xi’an Jiaotong University, Xi’an 710049, Shaanxi, People’s Republic of China

## Abstract

In the title coordination polymer, {[Co(CHO_2_)_2_(C_20_H_14_N_4_)(H_2_O)_2_]·2H_2_O}_*n*_, the Co^II^ atom (site symmetry 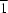) is coordinated by two formate O atoms, two water O atoms and two N atoms from two 1,4-bis­(1*H*-benzimidazol-1-yl)benzene ligands (*L*), resulting in a distorted *trans*-CoN_2_O_4_ octa­hedral coordin­ation environment. The complete *L* ligand is generated by crystallographic inversion symmetry and serves to bridge the cobalt ions into a chain propagating in [1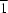 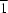]. The dihedral angle between the central benzene ring and the imidazole ring system is 38.48 (12)°. O—H⋯O hydrogen bonds involving both the coordinated and uncoordinated water mol­ecules occur and help to link the chains together.

## Related literature

For background to coordination polymers containing imidazole-derived ligands, see: Li *et al.* (2009 ▶, 2011 ▶).
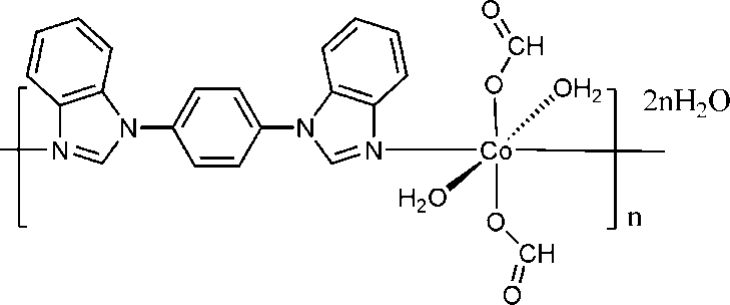


## Experimental

### 

#### Crystal data

[Co(CHO_2_)_2_(C_20_H_14_N_4_)(H_2_O)_2_]·2H_2_O
*M* *_r_* = 531.38
Triclinic, 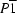

*a* = 7.497 (4) Å
*b* = 9.136 (5) Å
*c* = 9.443 (7) Å
α = 78.289 (19)°
β = 77.858 (19)°
γ = 67.72 (2)°
*V* = 579.6 (6) Å^3^
*Z* = 1
Mo *K*α radiation
μ = 0.80 mm^−1^
*T* = 293 K
0.22 × 0.20 × 0.18 mm

#### Data collection

Rigaku Mercury CCD area-detector diffractometer
Absorption correction: multi-scan (*CrystalClear*; Rigaku/MSC, 2005 ▶) *T*_min_ = 0.839, *T*_max_ = 0.867
4958 measured reflections
2012 independent reflections
1910 reflections with *I* > 2σ(*I*)
*R*_int_ = 0.026

#### Refinement

*R*[*F* ^2^ > 2σ(*F* ^2^)] = 0.038
*wR*(*F* ^2^) = 0.106
*S* = 1.10
2012 reflections
162 parameters
H-atom parameters constrained
Δρ_max_ = 1.08 e Å^−3^
Δρ_min_ = −0.46 e Å^−3^

### 

Data collection: *CrystalClear* (Rigaku/MSC, 2005 ▶); cell refinement: *CrystalClear*; data reduction: *CrystalClear*; program(s) used to solve structure: *SHELXS97* (Sheldrick, 2008 ▶); program(s) used to refine structure: *SHELXL97* (Sheldrick, 2008 ▶); molecular graphics: *SHELXTL* (Sheldrick, 2008 ▶); software used to prepare material for publication: *SHELXTL*.

## Supplementary Material

Crystal structure: contains datablock(s) I, global. DOI: 10.1107/S160053681105505X/hb6531sup1.cif

Structure factors: contains datablock(s) I. DOI: 10.1107/S160053681105505X/hb6531Isup2.hkl

Additional supplementary materials:  crystallographic information; 3D view; checkCIF report

## Figures and Tables

**Table 1:** Selected bond lengths (Å)

Co1—O1	2.1110 (19)
Co1—N1	2.136 (2)
Co1—O1*W*	2.1451 (19)

**Table 2:** Hydrogen-bond geometry (Å, °)

*D*—H⋯*A*	*D*—H	H⋯*A*	*D*⋯*A*	*D*—H⋯*A*
O1*W*—H1*A*⋯O2*W*^i^	0.83	1.94	2.759 (4)	170
O1*W*—H1*B*⋯O2^i^	0.90	1.83	2.691 (4)	159
O2*W*—H2*A*⋯O1^ii^	0.98	2.01	2.837 (4)	141
O2*W*—H2*B*⋯O2^iii^	0.88	1.89	2.766 (4)	170

Symmetry codes: (i) 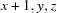; (ii) 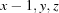; (iii) 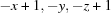.

**Fig. 1.**
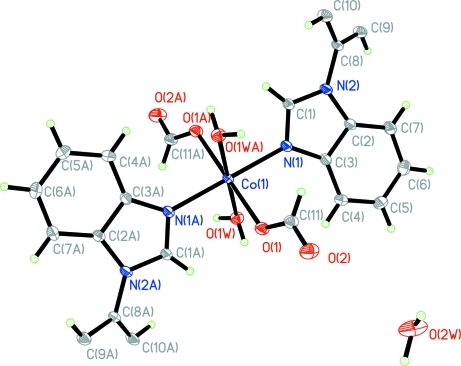
The molecular structure of (I). Displacement ellipsoids are drawn at the 30% probability level and H atoms are shown as small spheres of arbitrary radius.

**Fig. 2.**
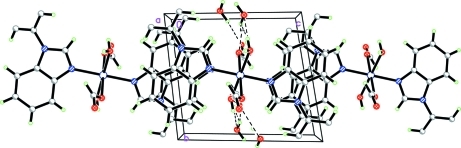
The crystal packing for (I).

## supplementary crystallographic information

### Comment

Imidazole has been extensively used in crystal engineering, and a large number of imidazole-containing flexible ligands have been extensively studied. However, to our knowledge, the research on imidazole ligands bearing rigid spacers is still less developed (Li *et al.*, 2009; Li *et al.*, 2011). For the title compound, the geometry of the Co^II^ ion is bound by two benzoimidazole rings of individual L ligands, two water molecules and two formate ions forming a slightly distorted octahedral coordination environment(Fig. 1). Notably, as shown in Fig. 2, the six-coordinate Co^II^ center is bridged by the ligand L to form an infinite one-dimensional architecture.

### Experimental

A mixture of CH_3_OH and H_2_O (1:1, 8 ml), as a buffer layer, was carefully layered over a solution of Co(HCO_2_)_2_ in H_2_O (6 ml). Then a solution of 1,4-di(1*H*-benzimidazol-1-yl)benzene (L, 0.06 mmol) in CH_3_OH (6 ml) was layered over the buffer layer, and the resultant reaction was left to stand at room temperature. After *ca* three weeks, purple blocks appeared at the boundary. Yield: ~21% (based on L).

### Refinement

C-bound H atoms were positioned geometrically and refined in the riding-model approximation, with C—H = 0.93Å and *U*_iso_(H) = 1.2*U*_eq_ (C).

### Crystal data

**Table:**

[Co(CHO_2_)_2_(C_20_H_14_N_4_)(H_2_O)_2_]·2H_2_O	*Z* = 1
*M**_r_* = 531.38	*F*(000) = 275
Triclinic, *P*1	*D*_x_ = 1.522 Mg m^−^^3^
Hall symbol: -P 1	Mo *K*α radiation, λ = 0.71073 Å
*a* = 7.497 (4) Å	Cell parameters from 6325 reflections
*b* = 9.136 (5) Å	θ = 2.9–53.8°
*c* = 9.443 (7) Å	µ = 0.80 mm^−^^1^
α = 78.289 (19)°	*T* = 293 K
β = 77.858 (19)°	Block, purple
γ = 67.72 (2)°	0.22 × 0.20 × 0.18 mm
*V* = 579.6 (6) Å^3^	

### Data collection

**Table:**

Rigaku Mercury CCD area-detector diffractometer	2012 independent reflections
Radiation source: fine-focus sealed tube	1910 reflections with *I* > 2σ(*I*)
graphite	*R*_int_ = 0.026
Detector resolution: 9 pixels mm^-1^	θ_max_ = 25.0°, θ_min_ = 2.2°
ω scans	*h* = −8→8
Absorption correction: multi-scan (*CrystalClear*; Rigaku/MSC, 2005)	*k* = −10→10
*T*_min_ = 0.839, *T*_max_ = 0.867	*l* = −11→11
4958 measured reflections	

### Refinement

**Table:**

Refinement on *F*^2^	Primary atom site location: structure-invariant direct methods
Least-squares matrix: full	Secondary atom site location: difference Fourier map
*R*[*F*^2^ > 2σ(*F*^2^)] = 0.038	Hydrogen site location: inferred from neighbouring sites
*wR*(*F*^2^) = 0.106	H-atom parameters constrained
*S* = 1.10	*w* = 1/[σ^2^(*F*_o_^2^) + (0.0635*P*)^2^ + 0.3613*P*] where *P* = (*F*_o_^2^ + 2*F*_c_^2^)/3
2012 reflections	(Δ/σ)_max_ < 0.001
162 parameters	Δρ_max_ = 1.08 e Å^−^^3^
0 restraints	Δρ_min_ = −0.46 e Å^−^^3^

### Special details

**Table:**

Geometry. All e.s.d.'s (except the e.s.d. in the dihedral angle between two l.s. planes) are estimated using the full covariance matrix. The cell e.s.d.'s are taken into account individually in the estimation of e.s.d.'s in distances, angles and torsion angles; correlations between e.s.d.'s in cell parameters are only used when they are defined by crystal symmetry. An approximate (isotropic) treatment of cell e.s.d.'s is used for estimating e.s.d.'s involving l.s. planes.
Refinement. Refinement of *F*^2^ against ALL reflections. The weighted *R*-factor *wR* and goodness of fit *S* are based on *F*^2^, conventional *R*-factors *R* are based on *F*, with *F* set to zero for negative *F*^2^. The threshold expression of *F*^2^ > σ(*F*^2^) is used only for calculating *R*-factors(gt) *etc*. and is not relevant to the choice of reflections for refinement. *R*-factors based on *F*^2^ are statistically about twice as large as those based on *F*, and *R*- factors based on ALL data will be even larger.

### Fractional atomic coordinates and isotropic or equivalent isotropic displacement parameters (Å^2^)

**Table:**

	*x*	*y*	*z*	*U*_iso_*/*U*_eq_	
Co1	1.0000	0.5000	0.5000	0.02012 (17)	
O1W	1.2375 (2)	0.3098 (2)	0.58992 (19)	0.0295 (4)	
O2W	0.1838 (4)	0.0285 (3)	0.5850 (3)	0.0609 (7)	
O1	0.8818 (2)	0.3208 (2)	0.5127 (2)	0.0293 (4)	
O2	0.6238 (3)	0.2504 (2)	0.5388 (3)	0.0463 (5)	
N1	0.8569 (3)	0.5511 (2)	0.7146 (2)	0.0265 (4)	
N2	0.7050 (3)	0.7105 (2)	0.8865 (2)	0.0275 (5)	
C1	0.7791 (4)	0.6984 (3)	0.7437 (3)	0.0292 (5)	
H1	0.7751	0.7864	0.6731	0.035*	
C2	0.7396 (3)	0.5554 (3)	0.9577 (3)	0.0262 (5)	
C3	0.8340 (3)	0.4566 (3)	0.8486 (3)	0.0243 (5)	
C4	0.8857 (4)	0.2915 (3)	0.8831 (3)	0.0325 (6)	
H4	0.9466	0.2244	0.8117	0.039*	
C5	0.8430 (4)	0.2314 (3)	1.0271 (3)	0.0414 (7)	
H5	0.8772	0.1216	1.0531	0.050*	
C6	0.7500 (5)	0.3310 (4)	1.1347 (3)	0.0450 (7)	
H6	0.7243	0.2857	1.2307	0.054*	
C7	0.6952 (4)	0.4944 (3)	1.1030 (3)	0.0374 (6)	
H7	0.6319	0.5606	1.1748	0.045*	
C8	0.6007 (3)	0.8580 (3)	0.9448 (3)	0.0257 (5)	
C9	0.6227 (4)	0.8724 (3)	1.0825 (3)	0.0306 (5)	
H9	0.7047	0.7868	1.1376	0.037*	
C10	0.4786 (4)	0.9844 (3)	0.8624 (3)	0.0319 (6)	
H10	0.4644	0.9732	0.7701	0.038*	
C11	0.7062 (4)	0.3410 (3)	0.5497 (3)	0.0295 (5)	
H11	0.6286	0.4339	0.5897	0.035*	
H1B	1.3579	0.3142	0.5608	0.044*	
H1A	1.2358	0.2205	0.5863	0.044*	
H2B	0.2325	−0.0572	0.5401	0.105 (17)*	
H2A	0.0565	0.0927	0.5551	0.091 (14)*	

### Atomic displacement parameters (Å^2^)

**Table:**

	*U*^11^	*U*^22^	*U*^33^	*U*^12^	*U*^13^	*U*^23^
Co1	0.0216 (3)	0.0196 (3)	0.0178 (3)	−0.00541 (17)	0.00065 (16)	−0.00702 (16)
O1W	0.0255 (8)	0.0273 (9)	0.0346 (10)	−0.0076 (7)	−0.0052 (7)	−0.0045 (7)
O2W	0.0647 (15)	0.0300 (11)	0.094 (2)	−0.0096 (10)	−0.0345 (14)	−0.0111 (12)
O1	0.0243 (9)	0.0274 (9)	0.0365 (10)	−0.0089 (7)	−0.0001 (7)	−0.0098 (7)
O2	0.0294 (10)	0.0370 (11)	0.0755 (16)	−0.0147 (9)	−0.0014 (10)	−0.0148 (10)
N1	0.0332 (11)	0.0229 (10)	0.0193 (10)	−0.0060 (8)	0.0009 (8)	−0.0061 (8)
N2	0.0362 (11)	0.0217 (10)	0.0195 (10)	−0.0044 (8)	0.0003 (8)	−0.0076 (8)
C1	0.0421 (14)	0.0226 (12)	0.0182 (11)	−0.0081 (10)	0.0019 (10)	−0.0055 (9)
C2	0.0279 (12)	0.0229 (12)	0.0239 (12)	−0.0047 (10)	−0.0010 (9)	−0.0060 (9)
C3	0.0241 (11)	0.0249 (11)	0.0214 (12)	−0.0059 (9)	−0.0015 (9)	−0.0052 (9)
C4	0.0347 (13)	0.0232 (12)	0.0349 (14)	−0.0053 (10)	−0.0002 (11)	−0.0084 (10)
C5	0.0518 (17)	0.0252 (13)	0.0386 (16)	−0.0097 (12)	−0.0016 (13)	0.0015 (11)
C6	0.0608 (19)	0.0378 (15)	0.0274 (15)	−0.0158 (14)	0.0018 (13)	0.0036 (12)
C7	0.0493 (16)	0.0341 (14)	0.0223 (13)	−0.0104 (12)	0.0026 (11)	−0.0061 (11)
C8	0.0317 (12)	0.0215 (11)	0.0212 (12)	−0.0061 (9)	0.0013 (9)	−0.0089 (9)
C9	0.0374 (14)	0.0256 (12)	0.0241 (12)	−0.0037 (10)	−0.0073 (10)	−0.0047 (10)
C10	0.0434 (14)	0.0296 (13)	0.0196 (12)	−0.0060 (11)	−0.0058 (10)	−0.0089 (10)
C11	0.0267 (13)	0.0265 (12)	0.0337 (14)	−0.0067 (10)	−0.0042 (10)	−0.0058 (10)

### Geometric parameters (Å, °)

**Table:**

Co1—O1^i^	2.1110 (19)	C2—C7	1.394 (4)
Co1—O1	2.1110 (19)	C2—C3	1.402 (3)
Co1—N1	2.136 (2)	C3—C4	1.393 (4)
Co1—N1^i^	2.136 (2)	C4—C5	1.378 (4)
Co1—O1W^i^	2.1451 (19)	C4—H4	0.9300
Co1—O1W	2.1451 (19)	C5—C6	1.394 (4)
O1W—H1B	0.8998	C5—H5	0.9300
O1W—H1A	0.8288	C6—C7	1.375 (4)
O2W—H2B	0.8823	C6—H6	0.9300
O2W—H2A	0.9779	C7—H7	0.9300
O1—C11	1.240 (3)	C8—C10	1.382 (4)
O2—C11	1.236 (3)	C8—C9	1.383 (3)
N1—C1	1.309 (3)	C9—C10^ii^	1.384 (4)
N1—C3	1.398 (3)	C9—H9	0.9300
N2—C1	1.355 (3)	C10—C9^ii^	1.384 (3)
N2—C2	1.391 (3)	C10—H10	0.9300
N2—C8	1.432 (3)	C11—H11	0.9300
C1—H1	0.9300		
			
O1^i^—Co1—O1	180.000 (1)	N2—C2—C3	105.4 (2)
O1^i^—Co1—N1	88.37 (8)	C7—C2—C3	122.2 (2)
O1—Co1—N1	91.63 (8)	C4—C3—N1	130.5 (2)
O1^i^—Co1—N1^i^	91.63 (8)	C4—C3—C2	120.3 (2)
O1—Co1—N1^i^	88.37 (8)	N1—C3—C2	109.2 (2)
N1—Co1—N1^i^	180.0	C5—C4—C3	117.4 (2)
O1^i^—Co1—O1W^i^	84.83 (8)	C5—C4—H4	121.3
O1—Co1—O1W^i^	95.17 (8)	C3—C4—H4	121.3
N1—Co1—O1W^i^	89.43 (8)	C4—C5—C6	121.8 (3)
N1^i^—Co1—O1W^i^	90.57 (8)	C4—C5—H5	119.1
O1^i^—Co1—O1W	95.17 (8)	C6—C5—H5	119.1
O1—Co1—O1W	84.83 (8)	C7—C6—C5	121.9 (3)
N1—Co1—O1W	90.57 (8)	C7—C6—H6	119.1
N1^i^—Co1—O1W	89.43 (8)	C5—C6—H6	119.1
O1W^i^—Co1—O1W	180.00 (9)	C6—C7—C2	116.5 (3)
Co1—O1W—H1B	117.7	C6—C7—H7	121.7
Co1—O1W—H1A	112.5	C2—C7—H7	121.7
H1B—O1W—H1A	111.6	C10—C8—C9	120.7 (2)
H2B—O2W—H2A	107.9	C10—C8—N2	119.4 (2)
C11—O1—Co1	123.68 (16)	C9—C8—N2	119.8 (2)
C1—N1—C3	105.12 (19)	C8—C9—C10^ii^	119.3 (2)
C1—N1—Co1	120.75 (16)	C8—C9—H9	120.3
C3—N1—Co1	133.95 (16)	C10^ii^—C9—H9	120.3
C1—N2—C2	106.57 (19)	C8—C10—C9^ii^	119.9 (2)
C1—N2—C8	124.6 (2)	C8—C10—H10	120.0
C2—N2—C8	128.7 (2)	C9^ii^—C10—H10	120.0
N1—C1—N2	113.7 (2)	O2—C11—O1	126.7 (2)
N1—C1—H1	123.2	O2—C11—H11	116.7
N2—C1—H1	123.2	O1—C11—H11	116.7
N2—C2—C7	132.4 (2)		
			
O1^i^—Co1—O1—C11	166 (100)	Co1—N1—C3—C4	−6.5 (4)
N1—Co1—O1—C11	50.3 (2)	C1—N1—C3—C2	−0.2 (3)
N1^i^—Co1—O1—C11	−129.7 (2)	Co1—N1—C3—C2	174.71 (17)
O1W^i^—Co1—O1—C11	−39.3 (2)	N2—C2—C3—C4	−178.4 (2)
O1W—Co1—O1—C11	140.7 (2)	C7—C2—C3—C4	0.5 (4)
O1^i^—Co1—N1—C1	39.7 (2)	N2—C2—C3—N1	0.5 (3)
O1—Co1—N1—C1	−140.3 (2)	C7—C2—C3—N1	179.4 (2)
N1^i^—Co1—N1—C1	178 (100)	N1—C3—C4—C5	−179.6 (3)
O1W^i^—Co1—N1—C1	−45.2 (2)	C2—C3—C4—C5	−1.0 (4)
O1W—Co1—N1—C1	134.8 (2)	C3—C4—C5—C6	0.6 (4)
O1^i^—Co1—N1—C3	−134.6 (2)	C4—C5—C6—C7	0.2 (5)
O1—Co1—N1—C3	45.4 (2)	C5—C6—C7—C2	−0.8 (5)
N1^i^—Co1—N1—C3	3(100)	N2—C2—C7—C6	178.9 (3)
O1W^i^—Co1—N1—C3	140.6 (2)	C3—C2—C7—C6	0.4 (4)
O1W—Co1—N1—C3	−39.4 (2)	C1—N2—C8—C10	36.0 (4)
C3—N1—C1—N2	−0.3 (3)	C2—N2—C8—C10	−139.4 (3)
Co1—N1—C1—N2	−176.00 (16)	C1—N2—C8—C9	−144.2 (3)
C2—N2—C1—N1	0.6 (3)	C2—N2—C8—C9	40.4 (4)
C8—N2—C1—N1	−175.6 (2)	C10—C8—C9—C10^ii^	−0.3 (4)
C1—N2—C2—C7	−179.3 (3)	N2—C8—C9—C10^ii^	179.9 (2)
C8—N2—C2—C7	−3.3 (5)	C9—C8—C10—C9^ii^	0.3 (4)
C1—N2—C2—C3	−0.7 (3)	N2—C8—C10—C9^ii^	−179.9 (2)
C8—N2—C2—C3	175.3 (2)	Co1—O1—C11—O2	168.5 (2)
C1—N1—C3—C4	178.6 (3)		

Symmetry codes: (i) −*x*+2, −*y*+1, −*z*+1; (ii) −*x*+1, −*y*+2, −*z*+2.

### Hydrogen-bond geometry (Å, °)

**Table:**

*D*—H···*A*	*D*—H	H···*A*	*D*···*A*	*D*—H···*A*
O1W—H1A···O2W^iii^	0.83	1.94	2.759 (4)	170
O1W—H1B···O2^iii^	0.90	1.83	2.691 (4)	159
O2W—H2A···O1^iv^	0.98	2.01	2.837 (4)	141
O2W—H2B···O2^v^	0.88	1.89	2.766 (4)	170

Symmetry codes: (iii) *x*+1, *y*, *z*; (iv) *x*−1, *y*, *z*; (v) −*x*+1, −*y*, −*z*+1.
